# Human Metapneumovirus and Respiratory Syncytial Virus Disease in Children, Yemen

**DOI:** 10.3201/eid1209.060207

**Published:** 2006-09

**Authors:** Najla Al-Sonboli, Charles A. Hart, Nasher Al-Aghbari, Ahmed Al-Ansi, Omar Ashoor, Luis E. Cuevas

**Affiliations:** * Liverpool School of Tropical Medicine, Liverpool, United Kingdom;; † Sana'a University, Sana'a, Yemen;; ‡ University of Liverpool, Liverpool, United Kingdom;; § Al-Sabeen Hospital for Women and Children, Sana'a, Yemen

**Keywords:** Risk factors, children, disease severity, acute respiratory infections, human metapneumovirus, respiratory syncytial virus, Yemen, dispatch

## Abstract

Factors increasing the severity of respiratory infections in developing countries are poorly described. We report factors associated with severe acute respiratory illness in Yemeni children (266 infected with respiratory syncytial virus and 66 with human metapneumovirus). Age, indoor air pollution, and incomplete vaccinations were risk factors and differed from those in industrialized countries.

Acute respiratory infections (ARIs) are the main cause of childhood death worldwide (*1*). Respiratory syncytial virus (RSV) is most frequently implicated in childhood illness (*2*). Although factors predisposing to severe ARI caused by RSV are well known in industrialized countries, little information exists for developing countries. Infection with human metapneumovirus (HMPV) has clinical symptoms similar to those for RSV (*3*). Despite its frequency, little information exists on factors that predispose children to severe ARI caused by HMPV. We describe factors associated with severe ARI caused by RSV or HMPV in Yemeni children.

## The Study

Children <2 years of age with ARI attending Al-Sabeen Hospital, a reference hospital in Sana'a, Yemen, were enrolled in a study from October 2002 to May 2003. All children attending emergency and outpatient services between 8:00 a.m. and 1:00 a.m. with signs and symptoms of ARI were recruited independent of disease severity. Diagnosis was based on clinical signs, as suggested by the World Health Organization protocol for research on ARI (*4*). A total of 62% of the patients were hospitalized. Patients admitted at night were recruited the next morning.

Oxygen pressure (pO_2_) was measured by using pulse oximetry (Nonin Medical, Inc., Plymouth, MN, USA), and children were classified as having no or moderate (pO_2_ >88%) or severe (pO_2_<88%) hypoxia, as suggested for high altitudes (*5*), since Sana'a is 2,200 m above sea level. Nasopharyngeal aspirates were tested by using reverse transcription PCR, as previously reported (*6*).

Children with severe hypoxia caused by RSV were compared with children with RSV and no or moderate hypoxia. The same comparisons were used for children infected with HMPV. The χ^2^ and Student *t* tests were used, and values with p values <0.20 were entered into backward logistic regressions. Ethical approval was obtained from the ethics committees of the Liverpool School of Tropical Medicine and Al-Sabeen Hospital. Parents were interviewed after informed consent was obtained.

A total of 325 (54%) children were recruited from the emergency service, 235 (39%) from outpatient clinics and 41 (7%) from wards. RSV was identified in 266 (44%) and HMPV in 66 (11%) children, including 25 (4%) coinfected with RSV and HMPV. Two hundred thirty-two (87%) children with RSV, 46 (70%) with HMPV (p<0.01), and 22 (88%) coinfected with both viruses had severe hypoxia (Table). Among RSV-positive specimens, 171 (82%) of 208 were group A and 37 (18%) of 208 were group B. No association was seen between groups or genotypes and hypoxia.

**Table:** Characteristics of children with mild and severe hypoxia caused by infection with RSV and HMPV*

Characteristic	RSV hypoxia			HMPV hypoxia		
	Mild (n = 34)	Severe (n = 232)	p value	Mild (n = 20)	Severe (n = 46)	p value
Male	22 (65)	154 (66)	0.4	17 (85)	30 (65)	0.08
Mean (SD) age, mo.	7.9 (5.6)	4.2 (4.8)	<0.001	9.1 (5.9)	5.7 (5.4)	0.02
Preterm	1 (3)	11 (5)	0.5	1 (5)	3 (7)	0.6
Height-for-age Z scores <–2	13 (38)	73 (32)	0.2	12 (60)	19 (41.3)	0.08
Not exclusively breastfed	12 (35)	136 (59)	0.01	15 (75)	23 (50)	0.1
Incomplete vaccinations	7 (21)	178 (77)	<0.001	4 (20)	30 (65)	<0.001
Recurrent ARI	6 (18)	40 (17)	0.4	2 (10)	15 (33)	0.04
Recurrent wheeze	1 (3)	12 (5)	0.4	0 (0)	5 (11)	0.1
Eczema	1 (3)	11 (5)	0.5	1 (5)	1 (2)	0.5
Asthma	1 (3)	1 (0.4)	0.2	0	3 (7)	0.3
CHD	0	2 (1)	0.7	0	0	0.3
Family member with ARI	18 (53)	165 (71)	0.02	7 (35)	28 (61)	0.03
Family member with allergies	3 (9)	29 (13)	0.3	4 (20)	4 (9)	0.1
Family member with asthma	5 (15)	24 (10)	0.2	1 (5)	5 (11)	0.4
Family member with eczema	3 (9)	11 (5)	0.2	1 (5)	5 (11)	0.4
Smoker at home	9 (27)	120 (52)	0.002	10 (50)	28 (61)	0.2
Indoor animals	9 (27)	97 (42)	0.04	4 (20)	15 (33)	0.2
Cows	1 (3)	52 (22)	0.003	0 (0)	9 (20)	0.02
Goats	6 (18)	69 (30)	0.07	1 (5)	7 (15)	0.2
Chicken	3 (9)	39 (17)	0.1	2 (10)	7 (15)	0.4
Cats	3 (9)	26 (11)	0.4	3 (15 )	7 (15)	0.6
Dogs	0	8 (3)	0.3	0	0	0.2
Donkeys	0	32 (10)	0.03	0	4 (9)	0.2
Outdoor animals	2 (6)	88 (38)	<0.001	1 (5)	14 (30)	0.01
Private source of water	11 (32)	138 (60)	0.002	5 (25)	22 (47)	0.04
Cooking fuel other than gas	2 (6)	128 (55)	<0.001	2 (10)	23 (50)	0.001

*Values are no. (%) unless otherwise indicated. RSV, respiratory syncytial virus; HMPV, human metapneumovirus; SD, standard deviation; ARI, acute respiratory illness; CHD, congenital heart disease.

Children <3 months of age were more likely to have severe hypoxia if they were infected with both viruses (Figure). Children with severe RSV hypoxia were more likely to have relatives with ARI, and this factor plus a history of recurrent respiratory infections were risk factors for HMPV hypoxia. Personal or family histories of atopy, prematurity, or chronic lung or congenital heart diseases were not associated with ARI severity caused by RSV or HMPV.

**Figure:**
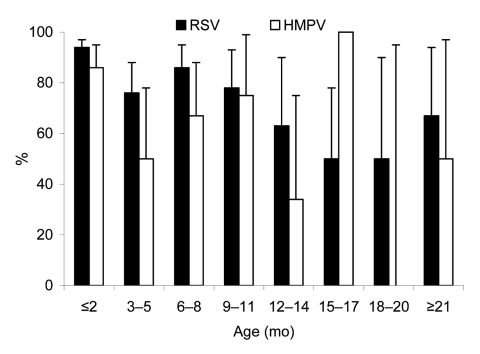
Proportion of children with severe infection with respiratory syncytial virus (RSV) and human metapneumovirus (HMPV), by age, Yemen. Error bars show 95% confidence intervals.

Among household characteristics, the presence of animals (cows and donkeys), a person in the house smoking, using private water sources, and cooking with fuels other than gas were more frequent for children with severe RSV hypoxia. Only the presence of cows, private water, and cooking fuels other than gas were associated with severe HMPV hypoxia.

Multivariate analysis showed that age <3 months and using cooking fuels other than gas were risk factors for severe RSV hypoxia (adjusted odds ratio [AOR] 3.4, 95% confidence interval [CI] 1.3–8.7 and AOR 10.3, 95% CI 2.2–48) and HMPV hypoxia (AOR 14.2, 95% CI 3.1–65 and AOR 13.1, 95% CI 2.2–78), while incomplete vaccinations (AOR 4.5, 95% CI 1.7–12) and smoking (AOR 3.8, 95% CI 1.5–9.8) were associated with severe RSV hypoxia but not HMPV hypoxia. Conversely, a history of recurrent ARI (AOR 13, 95% CI 2.0–84) was associated with severe HMPV hypoxia but not with RSV hypoxia.

## Conclusions

Identification of factors that increase the severity of ARI may affect health policies. However, little information is available about such factors in developing countries. Young children are especially susceptible to severe ARI, and our findings confirm that in a hospital setting age is a factor for both severe RSV and severe HMPV ARI. Prematurity and congenital heart and chronic lung diseases, which have been associated with increased risk for hospitalization for RSV and HMPV infections, were not risk factors. Since most Yemeni children are born at home, with limited access to health services, these children are underrepresented in our sample. We did not identify malnutrition as a risk factor for RSV or HMPV infection. Infection with RSV was more frequent and severe in well-nourished children in Nigeria, The Gambia, and Chile (*7*), but this finding was not confirmed by other investigators (*8*), and it is still contentious.

We found no association of the 2 viral infections with atopy. Although no information exists about their role in infection with HMPV, increased levels of common allergens within households had no effect on RSV infection severity in the United States (*9*). However, Gambian mothers of children hospitalized with RSV infections reported asthma more frequently in their children than mothers of nonhospitalized children (*10*). The role of atopy in development of severe RSV or HMPV infections in developing countries needs further elucidation.

Several reports have suggested that coinfections with RSV and HMPV increase disease severity, but we did not find such an interaction. Although we cannot exclude coinfection with bacterial or other viral agents, for which RSV or HMPV may increase disease severity, our findings are similar to reports from the Mediterranean region (*11**,**12*). This finding suggests that HMPV strains vary with location and time, and certain strains increase disease severity.

Children with severe hypoxia were less likely to be vaccinated, which is a likely indicator of poverty because poor parents in Yemen only use health services if their children are ill and are less likely to have their children vaccinated. In industrialized countries, poverty is associated with a higher incidence of RSV, and risk factors for infection with RSV are more likely to occur simultaneously (*13*).

We also observed that other household characteristics, such as cooking fuel and water, proximity to animals and relatives with ARI, or smoking increased the risk for hypoxia. Although we did not quantify air pollution within households, traditional mud stoves are built at ground level, use wood or dung, and generate large amounts of fumes, which blacken adjacent walls. Exposure to this pollution is associated with severe ARI. Parental and maternal smoking during pregnancy, indoor pollution, and presence of pets are also risk factors in industrialized countries (*14**,**15*). However, in The Gambia the relationship between severe RSV infection and frequency of cooking was inverse (*10*), which may reflect the lower socioeconomic status of mothers who cook frequently; this finding needs to be explored further. Finally, as a hospital-based study, our study had a selection bias toward children with severe ARI, and community-based studies might find different risk factors than those reported here.

In conclusion, age <3 months, incomplete vaccinations, persons smoking in the house, and using cooking fuels other than gas were associated with an increased risk for severe RSV hypoxia. Similarly, age <3 months, using cooking fuels other than gas, and recurrent ARI were associated with severe HMPV hypoxia. Interventions to eliminate air pollution in households may reduce the severity of RSV and HMPV infections in developing countries, and further studies should be encouraged.
